# Investigating the Role of Leukocyte Telomere Length in Treatment-Resistant Depression and in Response to Electroconvulsive Therapy

**DOI:** 10.3390/jpm11111100

**Published:** 2021-10-27

**Authors:** Claudia Pisanu, Erika Vitali, Anna Meloni, Donatella Congiu, Giovanni Severino, Raffaella Ardau, Caterina Chillotti, Luigi Trabucchi, Marco Bortolomasi, Massimo Gennarelli, Alessandra Minelli, Alessio Squassina

**Affiliations:** 1 Department of Biomedical Science, Section of Neuroscience and Clinical Pharmacology, University of Cagliari, 09042 Cagliari, Italy; claudia.pisanu@unica.it (C.P.); anna.meloni@unica.it (A.M.); dcongiu@unica.it (D.C.); severino@unica.it (G.S.); 2 Department of Molecular and Translational Medicine, University of Brescia, 25121 Brescia, Italy; e.vitali002@unibs.it (E.V.); massimo.gennarelli@unibs.it (M.G.); alessandra.minelli@unibs.it (A.M.); 3 Genetics Unit, IRCCS Istituto Centro San Giovanni di Dio Fatebenefratelli, 25125 Brescia, Italy; 4 Unit of Clinical Pharmacology, University Hospital Agency of Cagliari, 09123 Cagliari, Italy; ardau.raf@tiscali.it (R.A.); katine@tiscali.it (C.C.); 5 Psychiatric Hospital “Villa Santa Chiara”, 37142 Verona, Italy; luigitrabucchi@virgilio.it (L.T.); marcobortolomasi.vr@gmail.com (M.B.)

**Keywords:** telomere, ECT, treatment-resistance, accelerated aging, psychiatric disorders, mental disorders, GWAS, bipolar disorder, major depressive disorder, senescence

## Abstract

Psychiatric disorders seem to be characterized by premature cell senescence. However, controversial results have also been reported. In addition, the relationship between accelerated aging and treatment-resistance has scarcely been investigated. In the current study, we measured leukocyte telomere length (LTL) in 148 patients with treatment-resistant depression (TRD, 125 with major depressive disorder, MDD, and 23 with bipolar disorder, BD) treated with electroconvulsive therapy (ECT) and analyzed whether LTL was associated with different response profiles. We also compared LTL between patients with TRD and 335 non-psychiatric controls. For 107 patients for which genome-wide association data were available, we evaluated whether a significant overlap among genetic variants or genes associated with LTL and with response to ECT could be observed. LTL was negatively correlated with age (Spearman’s correlation coefficient = −0.25, *p* < 0.0001) and significantly shorter in patients with treatment-resistant MDD (Quade’s F = 35.18, *p* < 0.0001) or BD (Quade’s F = 20.84, *p* < 0.0001) compared to controls. Conversely, baseline LTL was not associated with response to ECT or remission. We did not detect any significant overlap between genetic variants or genes associated with LTL and response to ECT. Our results support previous findings suggesting premature cell senescence in patients with severe psychiatric disorders and suggest that LTL could not be a predictive biomarker of response to ECT.

## 1. Introduction

Mood disorders affect 5.4% of the general population representing a substantial socioeconomic burden and significantly impacting the patients’ quality of life [1,2,3]. The most common and severe mood disorders are Major Depressive Disorder (MDD) and Bipolar Disorder (BD), which affect 6% and 0.8–1.2% of the world population, respectively [4,5]. Mood disorders are associated with a reduced life expectancy compared to the general population (up to 20 years), and this is largely accounted for by a higher incidence of cardiovascular and metabolic disorders [6,7] and suicide [8,9,10].

In both disorders, pharmacological treatments represent the main approach. The treatment of BD is complex because different medications are needed for each phase of the disorder and may differ in the acute and in the maintenance phase [11]. The treatment of bipolar depression is a big challenge because there are few drugs with proven efficacy and the use of antidepressants is controversial [12,13,14], due to the risk for manic switch [15] and the higher risk of suicidal ideation [16,17]. Nevertheless, antidepressants, either in monotherapy or in combination with other psychotropic medications, constitute the treatment of first choice for MDD and for bipolar depression, although on average only about 40 to 60% of treated patients show a significant improvement of symptoms [18,19], with about one third of patients resistant to antidepressants [20]. Treatment-Resistant Depression (TRD) is defined as a failure to respond to two or more adequate trials of two or more different classes of antidepressants and to an adequate trial of a tricyclic (TCA) drug referred to Stage III of Thase and Rush Staging Method [21]. TRD concerns 10–30% of patients with MDD [19,22,23] and 75% of cases of unresolved morbidity of BD [24].

Electroconvulsive therapy (ECT) [25] is a non-pharmacological treatment indicated for TRD involving both unipolar and bipolar depression [26]. It is also recommended when there is an urgency of treatment (e.g., suicide risk) [27], for treatment-resistant schizophrenia [28] and catatonia [29]. ECT is based on an electrical stimulation of the brain with electrodes placed on the head of the patient under anesthesia; the electrical current generated triggers generalized seizures. Recent findings have also shown how ECT can induce antidepressant effects without inducing seizures [30], for example by changing sleep architecture [31]. The mechanism by which it determines an antidepressant effect is complex and not entirely clear [32]. ECT affects neurotransmission [33,34,35], induces changes in neuroendocrine [36] and neurotrophic factors [37,38,39,40], and also alters inflammatory mechanisms [41]. Some studies also showed that ECT can induce structural changes in the brain [42,43]. However, ECT can trigger side effects of different magnitude like headaches, nausea, prolonged seizures and, more seriously, the possibility of memory loss and cognitive side effects [44,45].

Although ECT is one of the most effective therapies for the management of TRD [46,47] because of its invasive nature and side effects, many studies tried to find parameters that can help predict the outcome of the treatment. Interestingly, one of the mechanisms by which ECT has been suggested to exert its antidepressant effects is a modification of inflammatory response [48,49]. A large body of data suggest that low grade inflammation might play a role in psychiatric disorders, and that the modulation of the inflammatory cascade could contribute to improving the symptomatology and the effectiveness of psychotropic medications [50,51,52]. The role of peripheral inflammation on functions of the central nervous system and on the etiopathogenesis of mood disorders is not well understood, but several hypotheses have been explored. Inflammatory processes have also shown to significantly contribute to accelerate cellular aging, mainly through acceleration of telomere shortening [53]. Telomere length (TL), a biomarker of aging widely investigated in psychiatric disorders, has been shown to be inversely correlated with levels of inflammation markers [53,54]. Telomeres are DNA-protein complexes located at the ends of eukaryotic chromosomes that play a crucial role in several physiological functions including maintaining structural integrity, three-dimensional architecture, DNA stability and prevention of uncontrolled replication [55]. Telomeres are progressively reduced in size (about 20–200 pairs of bases) with each replicative cycle [56]. This process continues until telomeres reach a critical minimum length that signals the end of proliferation, the beginning of senescence and the subsequent death by apoptosis of the cell [57,58]. This shortening is counteracted by the enzyme telomerase in cells in which this enzyme is active. However, telomerase seems to represent one of the many mechanisms involved in finely regulating telomere dynamics. TL is a heritable trait, with heritability estimated at 44–86% [59]. Genome-Wide Association Studies (GWAS) have identified a number of genetic variants associated with this trait, some of which are located in or near genes playing a crucial role in telomere regulation or DNA repair [59,60,61]. Interestingly, in a recent study, the minor allele of the rs2736100 single nucleotide polymorphism (SNP), located in the gene encoding for the catalytic subunit of telomerase (*TERT*), was associated with depression among those without experience of childhood adversity, as well as with the number of depressive episodes in patients with BD good responders to lithium [62].

Telomere shortening can also be accelerated by numerous factors such as oxidative stress, mutations of genes that play a key role in telomere biology, depletion of telomeric proteins [63], increased hypothalamic-pituitary-adrenal axis activity, decrease of some neurotrophic factors such as BDNF [64] and, as previously pointed out, inflammation. Leukocyte Telomere Length (LTL) can easily be measured from a non-invasive blood sampling and has been suggested to be related to the length of telomeres in other somatic cells [65].

Several studies investigated TL in patients with MDD and different results have been found, some of which showed that TL is shorter in patients than in non-psychiatric controls [66,67,68,69,70]. Similar findings have been reported in BD [67,71,72,73,74], and different studies showed that treatment with the mood stabilizer lithium correlated with longer LTL [75,76,77]. Fewer studies investigated differences in TL between responders and non-responders to antidepressant treatment. In a recent study, TL pre-treatment with SSRI was shorter in non-responders than in responders [78]. To date, only one study examined if TL could be a predictive marker of ECT response in patients with TRD, showing no correlation between TL and effectiveness of ECT treatment [79].

Here we present the findings of a retrospective study where we investigated the correlation between baseline LTL, genetic variants associated with LTL and response to ECT in a sample of patients with unipolar and bipolar TRD. Moreover, we explored differences in LTL between patients with TRD and non-psychiatric controls.

## 2. Results

### 2.1. Comparison of LTL between Patients with Treatment-Resistant Depression and Controls

**Figure 1:**
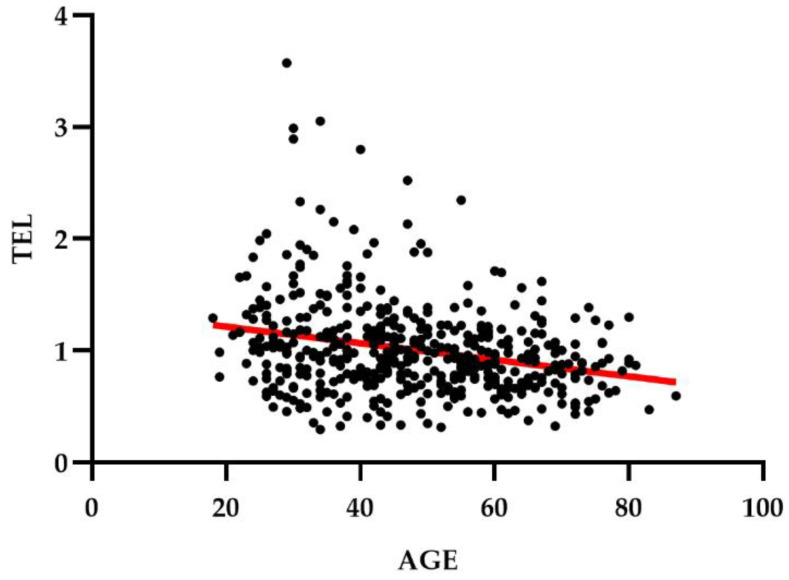
Flow-chart of the study.

**Figure 2:**
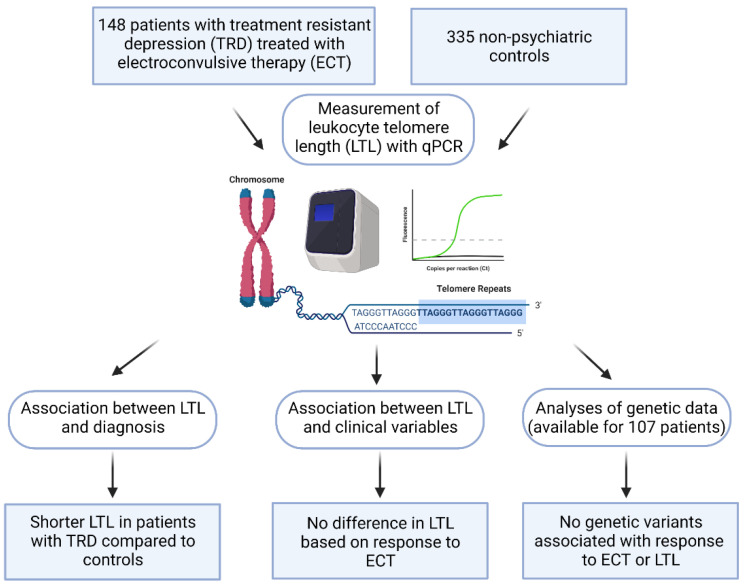
Negative correlation between LTL and age in the sample including 148 patients with TRD and 335 controls.

A flow-chart of the analyses is reported in Figure 1. Clinical data and LTL measurements were available for 149 patients with TRD. LTL from these patients was compared with LTL from 336 non-psychiatric controls. After exclusion of two outliers, analyses were conducted in a sample including 148 patients with TRD (125 with a diagnosis of MDD and 23 with a diagnosis of BD) and 335 controls (Table 1). LTL was negatively correlated with age (Spearman’s correlation coefficient = −0.25, *p* < 0.0001, Figure 2) and not associated with gender (U = 25,835, *p* = 0.079).

**Figure 3:**
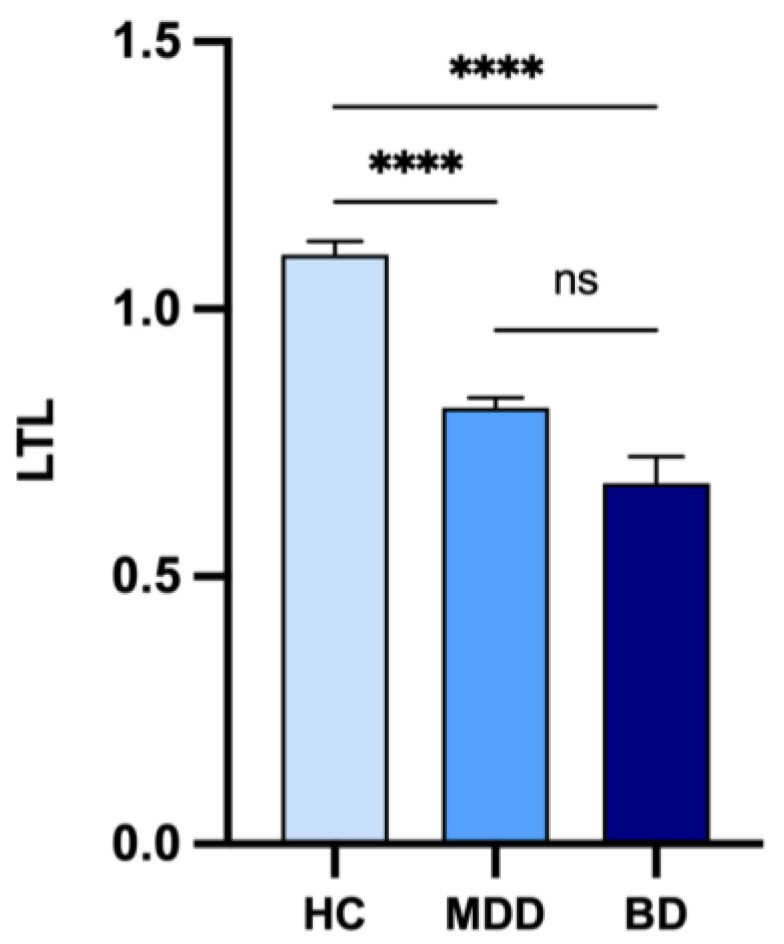
LTL in patients with treatment-resistant depression stratified based on diagnosis and controls. Barplots showing mean and standard error of the mean. **** *p* < 0.0001. Abbreviations: BD, bipolar disorder; HC, controls; MDD, major depressive disorder; ns, not significant.

LTL was shorter in patients with TRD compared to controls (U = 13,015, *p* < 0.0001, Table 2 and Figure 3). This result was significant also when adjusting for age using rank analysis of covariance (Quade’s F = 49.17, *p* < 0.0001). When stratifying patients with TRD based on their diagnosis (MDD and BD) using Kruskal-Wallis analysis with post-hoc tests with Dunn’s correction for multiple testing, both groups showed significantly shorter LTL compared to controls (MDD vs controls: U = 11.63, *p* < 0.0001, Quade’s F = 35.18, *p* < 0.0001; BD vs controls, U = 1.39, *p* < 0.0001, Quade’s F = 20.84, *p* < 0.0001). Similar results were obtained when repeating the analyses in a subsample of participants in which cases and controls were matched based on age (Supplementary Table S1).

### 2.2. Association between Baseline LTL and Response to ECT

In the whole sample of patients with TRD, baseline LTL was not significantly different between responders and non-responders at T1 or T2 or between remitters and non-remitters (Table 3) and was not correlated with the symptom improvement defined as the variation in the Montgomery and Asberg Depression Rating Scale (MADRS) between baseline and either T1 or T2. In addition, baseline LTL was not significantly correlated with MADRS scores at baseline or at any time point or with the number of ECT sessions (Table 3).

Similar results were obtained when stratifying the analyses based on psychiatric diagnoses or intake of the high-potency benzodiazepine clonazepam, which has been previously shown to negatively affect seizure quality [80] (Supplementary Tables S2 and S3).

### 2.3. Association between LTL and other Demographic or Clinical Variables

We observed a trend for negative correlation between LTL and body mass index (BMI, Spearman’s rho = −0.16, *p* = 0.067) and the association was significant after adjusting for age (partial correlation coefficient = −0.19, *p* = 0.027). Patients with comorbid personality disorders showed longer LTL in unadjusted analyses, but the association was not significant after adjusting for age (Mann Whitney’s U = 1456, *p* = 0.019; Model adjusted for age: Quade’s F = 2.36, *p* = 0.13). No other variable was significantly associated with LTL (Table 4).

We did not observe significant differences in baseline LTL based on intake of antidepressants, antipsychotics, mood stabilizers or benzodiazepines during ECT (Table 5).

### 2.4. Association between Genetic Variants, Response to ECT and LTL

Genome-wide genotyping data were available for 107 TRD patients. No SNP or gene was associated with either response to ECT or LTL at a genome-wide threshold (data not shown). Among 185,410 SNPs and 885 genes nominally associated with LTL, there was no significant enrichment for SNPs or genes nominally associated with response to ECT (Supplementary Table S4). Finally, among SNPs previously associated with LTL, we observed only few SNPs showing a nominal association with response to ECT (Supplementary Table S5). Specifically, the G allele of rs8105767 (closest gene: *ZNF208*) was associated with reduced symptom improvement at T1, the A allele of rs60160057 (*DCLK2*) with reduced improvement and lower odds of response at T2 and the C allele of rs7194734 (*MPHOSPH6*) was associated with increased LTL in the current sample but lower odds of response to ECT at T2.

## 3. Discussion

In the present study we showed no difference in LTL based on response to ECT in patients with TRD. This result is in accordance with the only available previous study that reported no association between whole blood TL and response to ECT, remission or cognitive side effects in a sample of 100 patients with severe depression in which improvement was assessed using the Hamilton rating scale for depression-24 [79]. Conversely, we observed significantly shorter LTL in patients with TRD compared to non-psychiatric controls. This finding is in line with previous studies showing shorter TL in patients with mood disorders [81].

We did not observe a correlation between LTL and severity of depression based on the MADRS score at baseline or at any time point evaluated. While this finding is in line with a previous work [82], a recent study on patients with late-life depression showed a negative correlation between LTL and severity of depressive symptoms measured with the Hamilton Depression Rating Scale [83]. These contrasting findings might be determined by several factors, including differences in the scales used to assess severity of symptoms and in demographic features (e.g., in our sample the median age of participants was 56 years, while the previous study only included patients with late-life depression). Moreover, all participants included in our study had a diagnosis of TRD, while the Hartmann et al., study [82] included MDD patients with no stratification based on resistance to treatments.

We also found that LTL was negatively correlated with BMI, after adjusting for age (Table 4). While this correlation could be partly mediated by increased levels of CRP [84], this relationship needs to be further investigated, since increased BMI has also been suggested to be associated with telomere attrition through non-inflammatory mechanisms [84].

In the analysis of GWAS data, we observed no significant enrichment between genetic variants associated with LTL and variants nominally associated with response to ECT. While LTL is a highly heritable trait, and shorter genetically predicted TL has been associated with increased risk of some disorders, such as coronary artery disease and other cardiovascular disorders [59,85], previous studies aiming to assess the presence of an enrichment between genetic variants associated with LTL and psychiatric phenotypes mostly yielded negative results [77,86,87].

The correlation between LTL and telomere dynamics in the central nervous system has been largely debated, but there is agreement in that TL in whole blood correlates with TL in most other tissues, including the brain [88]. While it is recognized that important differences exist in telomere biology and length in the different tissues of the human body, several studies suggest that LTL is significantly correlated with region-specific and total brain volume [89]. Interestingly, it has been suggested that shorter LTL is significantly associated with reduced size of the hippocampus [90]. The hippocampus is a key structure in the limbic system involved in multiple cognitive functions and has been reported to be significantly reduced in mood disorders [91].

Moreover, a correlation between reduce telomere length, accelerated brain aging and hippocampus has been postulated and supported to some extent by recent findings [92].

The aging brain is also characterized by altered neural circuits at different levels, but several studies support a key role of the thalamus and the pulvinar nucleus in particular [93,94]. A recent study showed that patients with schizophrenia had reduced functional brain connectivity in several areas compared to healthy controls, including the pulvinar nucleus, the hippocampus and the anterior cingulate cortex [95]. Interestingly, telomere length has been associated with wide-spread connectivity changes in the brain, particularly in the cingulum, a core component of the limbic system composed by a bundle of white fibers that connects to the frontal, parietal, middle temporal, and subcortical regions [96]. Moreover, some of the structural connectivity changes could partially explain the association between telomere length and executive function, a neuropsychological correlate highly reported to be impaired in mood disorder patients [97]. Overall, these data suggest that telomere shortening could contribute to the accelerated brain aging reported in mood disorder patients, and that this process might involve different regions as well as functional and structural connectivity processes in the brain.

Our results have to be interpreted in light of several limitations. The study included only a limited number of participants and the number of BD patients was particularly underpowered. Moreover, we applied a cross-sectional design, which did not allow exploring the causative role of telomere shortening or the correlation between longitudinal changes in telomere length and variations in the MADRS scores.

In conclusion, despite the aforementioned limitations, our study supports the hypothesis of accelerated cellular aging in mood disorders and suggests that neither telomere length nor genetic variants affecting it could constitute predictive biomarkers of response to ECT.

## 4. Materials and Methods

### 4.1. Participants

In accordance with the Diagnostic and Statistical Manual of Mental Disorders (Fourth Edition) classification system criteria, 149 TRD patients (of whom 23 BD) referred to the Psychiatric Hospital “Villa Santa Chiara,” Verona, Italy, were voluntarily enrolled in the study, which was approved by the local ethics committees (ethics committee of the province of Verona, No. 4997/09.11.01), and written informed consent was obtained. Diagnosis of unipolar or bipolar depression was confirmed using the Structured Clinical Interview for DSM-IV Axis 1 Disorders diagnostic structured interview. Exclusion criteria were the following: (i) mental retardation and cognitive disorders; (ii) a lifetime history of schizophrenic or schizoaffective; (iii) personality disorders, obsessive-compulsive disorder, or posttraumatic stress disorder as primary diagnosis; and (iv) comorbidity with eating disorders. All the patients were evaluated as treatment-resistant. Treatment-resistant depression was defined as at least the failure to respond to 2 or more adequate trials with 2 or more different classes of antidepressants and to an adequate trial of a tricyclic drug, referred to as stage III of Phase and Rush Staging Method [21]. All the patients were scheduled to undergo ECT. ECT was performed according to standard settings, with a bipolar brief pulse square wave and bilateral electrode placement. The ECT procedure has been described in detail elsewhere [40]. Illness severity and the outcome of ECT were assessed using the MADRS before the treatment (T0), the day after the end of ECT (T1) and about 1 month after its end (T2). Patients were considered as responders if the MADRS reduction was >50% at T1 or T2. In addition, symptom improvement at both time points was defined as the % variation (Delta) of MADRS score compared to baseline computed as (((T2 score or T1 score) − T0 score)/T0 score) × 100. Patients were considered remitters if they presented a MADRS score ≤ 10 at T2.

LTL measured in patients with TRD was compared with LTL from 336 healthy controls without any personal or family history of psychiatric conditions recruited at the Lithium Clinic of the Clinical Psychopharmacology Centre of the University Hospital of Cagliari. The research protocol was approved by the local Ethics Committee of the University of Cagliari, Italy. All participants signed informed written consent after a detailed description of the study procedures.

### 4.2. DNA Extraction

Genomic DNA was extracted from whole blood samples of 149 TRD patients using the Gentra Puregene Blood kit (Qiagen, Hilden, Germany), according to the manufacturer’s instructions. DNA quantification and quality evaluation were performed through spectrophotometric analysis (NanoDrop 2000, Thermo Scientific, Waltham, MA, USA). For non-psychiatric controls, genomic DNA was extracted from peripheral blood leukocytes using the salting-out method [98].

### 4.3. Measurements of Leukocyte Telomere Length with Quantitative PCR

Relative LTL was assessed according to the quantitative PCR (q-PCR) method as previously described [99]. Samples were processed in triplicates both for the telomere (Tel) and for the single-copy gene (hemoglobin-b, Hgb) using Platinum^®^ SYBR^®^ Green qPCR SuperMix-UDG w/ROX (Thermo Fisher Scientific, Waltham, MA, USA) on a StepOnePlus ™ Real-Time PCR System (Thermo Fisher Scientific). Primer sequences were as follows: Tel-1, 5′-GGTTTTTGAGGGTGAGGGTGAGGGTGAGGGTGAGGGT-3′, Tel-2, 5′-TCCCGACTATCCCTATCCCTATCCCTATCCCTATCCCTA-3′; Hgb1, 5′-GCTTCTGACACAACTGTGTTCACTAGC-3′, Hgb2, 5′-CACCAACTTCATCCACGTTCACC-3′. The PCR temperature conditions were 95 °C for 3 min followed by 28 cycles of 95 °C for 15 s and 60 °C for 1 min for Tel; 95 °C for 3 min followed by 32 cycles of 95 °C for 15 s and 60 °C for 1 min for Hgb. Specificity was assessed through the dissociation curve included in each plate. A control sample was included in each plate as a calibrator and LTL was calculated using the 2^−ΔΔCT^ method where ΔΔCT = ΔCT sample−ΔCT calibrator and ΔCT sample = CT Tel−CT Hgb.

### 4.4. Statistical Analysis

Normality of distribution was assessed using the Shapiro-Wilk test. Grubb’s test was used to identify outliers. The association between LTL and quantitative or categorical variables was assessed using Spearman’s correlation test or Mann-Whitney’s U test, respectively. In addition, we conducted analyses adjusted for age using partial correlation test or rank analyses of covariance (Quade’s test) to analyze the association between LTL and quantitative or categorical variables, respectively. Since patients and controls showed a significant difference in age, and this factor is known to be associated with LTL, we also repeated the analyses in a subsample of patients and controls matched using the Case Control Matching function in SPSS v. 26. A tolerance factor of 3 years was applied (this number was found to be the one able to minimize the loss of cases while still obtaining two subsamples of cases and controls that did not show a significant difference in age). Stratified analyses were conducted based on psychiatric diagnosis (BD or MDD) as well as based on treatment with the high-potency benzodiazepine clonazepam, that has been shown to negatively affect seizure quality in patients treated with ECT [80]. Analyses were conducted using GraphPad Prism v. 9 and SPSS v. 26. A flowchart of the study was created using BioRender (Figure 1).

### 4.5. Analysis of GWAS Data

For 107 TRD patients with LTL data, genome-wide genotyping data were available. Ninety-five patients were genotyped using Infinium Multi-Ethnic Genotyping Array, whereas twelve patients with the Infinium PsychArray-24 BeadChip (Illumina, San Diego, CA, USA). Quality control (QC) was performed for each dataset with PLINK v. 1.9 [100] in order to exclude SNPs with a minor allele frequency (MAF) < 0.05, a Hardy-Weinberg equilibrium (HWE) *p*-value < 1 × 10^−6^, and a call rate < 0.95; also, individuals with unusual heterozygosity (<0.20 or >0.40), a call rate < 0.99, cryptic relatedness (p_hat > 0.20) and sex discrepancy were removed. Imputation was performed based on the 1000 Genome data (Phase 3 Version 5) reference panel using Minimac 3 software through the Michigan Imputation Server, that provides genotype imputation service and an extensive quality controls check for all uploaded datasets [101]. After imputation, only biallelic variants with R^2^ ≥ 0.5, a genotype posterior probability (GP) > 0.9, MAF > 0.01 and in HWE (*p* > 1 × 10^−6^) were retained for statistical analyses. The single datasets were then merged together to generate one dataset including all cases. To reduce the batch effect due to multiple platforms, we removed from both datasets any SNPs showing significant association (FDR < 0.05) with the genotyping batch. Finally, we removed outlier individuals based on the inspection of the first 5 genotyping principal components (PCs) computed using PLINK v. 1.9.

The association between single nucleotide polymorphisms and categorical (response at T1 or T2) or quantitative variables (Delta MADRS % T0-T1, Delta MADRS % T0-T2 or LTL) was analyzed using binary logistic or linear regression, respectively, using PLINK v. 1.9 [100]. Analyses were also conducted at a gene-based level using MAGMA on the FUMA platform [102].

We also checked whether SNPs associated with LTL in previous studies were associated with LTL or response to ECT in our sample. To this aim, we selected 60 SNPs associated with LTL in previous publications [59,60,61], 22 of which were represented in our dataset (Supplementary Table S5). Using the hypergeometric test, we also checked if SNPs/genes nominally associated with LTL showed over-representation of SNPs/genes nominally associated with response to ECT.

## Figures and Tables

**Table 1:** Demographic characteristics of the sample.

	Patients with TRD (*n* = 148)	Controls (*n* = 335)	Statistics	*p*
Age, median (IQR)	56 (20)	43 (22)	14,125 ^a^	<0.0001
Gender (women, %)	67.6	53.1	8.76 ^b^	0.004

^a^ Mann-Whitney U; ^b^ = Pearson’s Chi-Square. Abbreviations: IQR, interquartile range.

**Table 2:** Comparison of LTL between patients with TRD and controls.

		Unadjusted Analyses		Analyses Adjusted for Age	
	LTL, median (IQR)	U	* p *	Quade’s F	* p *
Patients with TRD (*n* = 148)	0.77 (0.30)	13,015	<0.0001	49.17	<0.0001
Controls (*n* = 335)	1.03 (0.48)				

Abbreviations: corr. coeff., correlation coefficient; IQR, interquartile range; LTL, leukocyte telomere length; TRD, treatment-resistant depression.

**Table 3:** Comparison of baseline LTL in relation to ECT response.

		Unadjusted Analyses		Analyses Adjusted for Age	
	LTL, median (IQR)	U	*p*	Quade’s F	*p*
Responders at T1 (*n* = 119)	0.77 (0.30)	777	0.68	0.04	0.85
Non-Responders at T1 (*n* = 14)	0.85 (0.34)				
Responders at T2 (*n* = 65)	0.77 (0.34)	767	0.34	0.54	0.46
Non-Responders at T2 (*n* = 27)	0.88 (0.27)				
Remitters (*n* = 53)	0.75 (0.32)	769	0.18	1.50	0.23
Non-remitters (*n* = 35)	0.88 (0.29)				
	Median (IQR)	Spearman’s rho	*p*	partial corr. coeff.	*p*
Delta % MADRS T1-T0	75 (77)	−0.11	0.23	−0.12	0.17
Delta % MADRS T2-T0	78 (118)	−0.16	0.13	−0.18	0.10
MADRS scores at T0	33 (36)	0.03	0.73	0.03	0.76
MADRS scores at T1	8 (7)	0.11	0.20	0.13	0.15
MADRS scores at T2	7.5 (18)	0.17	0.12	0.17	0.12
Number of ECT sessions	7 (3)	0.03	0.71	0.02	0.79

Abbreviations: corr. coeff., correlation coefficient; IQR, interquartile range; LTL, leukocyte telomere length; MADRS, Montgomery Asberg Depression rating scale.

**Table 4:** Association between LTL and other demographic or clinical variables.

	Unadjusted Analyses		Analyses Adjusted for Age	
Variable	Statistics	*p*	Statistics	*p*
Years of education, median (IQR): 8 (8)	0.14 ^a^	0.10	0.01 ^b^	0.90
BMI, median (IQR): 26.4 (6.7)	−0.16 ^a^	0.067	**−0.19 ^b^**	**0.027**
Psychotic symptoms (70.9%)	2162 ^c^	0.687	0.09 ^d^	0.76
Smoking (35.1%)	2405 ^c^	0.72	0.24 ^d^	0.63
History of substance abuse (5.1%)	435 ^c^	0.85	0.27 ^d^	0.60
Comorbid alcohol abuse (2.7%)	244 ^c^	0.60	0.10 ^d^	0.75
Comorbid anxiety disorders (27.7%)	2030 ^c^	0.48	1.64 ^d^	0.20
Comorbid personality disorders (23.6%)	**1456** ^c^	**0.019**	2.36 ^d^	0.13
Comorbid cardiometabolic disorders (27.0%)	2055 ^c^	0.65	1.74 ^d^	0.19

^a^ Spearman’s rho; ^b^ partial correlation coefficient; ^c^ Mann-Whitney’s U; ^d^ Quade’s F Abbreviations: IQR, interquartile range.

**Table 5:** Association between LTL and medication intake.

	Unadjusted Analyses		Analyses Adjusted for Age	
Variable	U	*p*	Quade’s F	*p*
Antipsychotics (76.2%)	1730	0.30	0.56	0.46
Antidepressants (95.2%)	378	0.31	0.11	0.19
Mood stabilizers (15.0%)	1258	0.53	0.15	0.70
Benzodiazepines (87.1%)	1150	0.34	1.05	0.31

## Data Availability

Data available upon request from the authors.

## Supplementary Materials

The following are available online at www.mdpi.com/article/10.3390/jpm11111100/s1, Table S1: Comparison of LTL between patients with TRD and controls in a subsample of patients and controls matched for age; Table S2: Comparison of LTL between responders and non-responders to ECT in patients with TRD stratified based on psychiatric diagnosis; Table S3: Comparison of LTL between responders and non-responders to ECT in patients with TRD stratified based on intake of clonazepam; Table S4: Assessment of overrepresentation between SNPs and genes associated with response to ECT and LTL; Table S5: Association between SNP previously associated with LTL and response to ECT.
