# Supplementary material for: Investigating the Role of Leukocyte Telomere Length in Treatment-Resistant Depression and in Response to Electroconvulsive Therapy
Source: J Pers Med. 2021 Oct 27;11(11):1100. doi: 10.3390/jpm11111100 (PMC8622097; doi:10.3390/jpm11111100)
Supplement: Supplementary file 1 [file jpm-11-01100-s001.zip › jpm-1419379-supplementary.pdf]

Supplementary Table S1. Comparison of LTL between patients with TRD and controls in a subsample of patients and controls matched for age.

		Unadjusted analyses		Analyses adjusted for age	
	LTL, median (IQR)	U	p	Quade's F	p
Patients with TRD (n=147)	0.77 (0.30)	**5,567**	**< 0.0001**	**58.92**	**< 0.0001**
Controls (n=147)	1.03 (0.48)				
Patients with MDD (n=121)	0.79 (0.28)	**4,698**	**< 0.0001**	**47.91**	**< 0.0001**
Controls (n=147)	1.03 (0.48)				
Patients with BD (n=26)	0.70 (0.45)	**869**	**< 0.0001**	**22.81**	**< 0.0001**
Controls (n=147)	1.03 (0.48)				

LTL was compared between a subsample of 147 patients with TRD (median age: 56 years, IQR = 19 years) and 147 controls (median age: 54 years, IQR: 20 years) who were matched based on age using the Case Control Matching function in SPSS v. 26. A tolerance factor of 3 years was applied in order to minimize the loss of cases while still obtaining two groups of cases and controls that did not show a statistically significant difference in age (Mann Whitney's U = 9610, p = 0.10).

Abbreviations: IQR, interquartile range; LTL, leukocyte telomere length; TRD, treatment-resistant depression.

Supplementary Table S2. Comparison of LTL between responders and non-responders to ECT in patients with TRD stratified based on psychiatric diagnosis.

		Unadjusted analyses		Analyses adjusted for age	
	LTL, median (IQR)	U	p	Quade's F	p
**Patients with MDD**					
Responders at T1 (n=102)	0.79 (0.27)	598	0.57	0.30	0.59
Non-Responders at T1 (n=13)	0.86 (0.25)				
Responders at T2 (n=59)	0.77 (0.31)	598	0.17	0.94	0.34
Non-Responders at T2 (n=25)	0.89 (0.24)				
Remitters (n=47)	0.75 (0.30)	605	0.10	1.93	0.17
Non remitters (n=33)	0.88 (0.26)				
**Patients with BD**					
Responders at T1 (n=17)	0.65 (0.33)	5	0.67	0.39	0.54
Non-Responders at T1 (n=1)	-				
Responders at T2 (n=6)	0.76 (0.44)	3	0.43	0.47	0.52
Non-Responders at T2 (n=2)	0.60 (-)				
Remitters (n=6)	0.76 (0.44)	3	0.43	0.47	0.52
Non remitters (n=2)	0.60 (-)				
	Median (IQR)	Spearman's rho	p	partial corr. coeff.	p
**Patients with MDD**					
Delta % MADRS T1-T0	75.00 (21.22)	-0.13	0.15	-0.15	0.11
Delta % MADRS T2-T0	74.08 (55.75)	-0.19	0.09	-0.18	0.12
**Patients with BD**					
Delta % MADRS T1-T0	79.22 (31.16)	0.15	0.56	0.18	0.50
Delta % MADRS T2-T0	94.17 (41.81)	0.22	0.60	0.19	0.69

Abbreviations: BD, bipolar disorder; corr. coeff, correlation coefficient; IQR, interquartile range; LTL, leukocyte telomere length; MADRS, Montgomery Asberg Depression rating scale; MDD, major depressive disorder.

Supplementary Table S3. Comparison of LTL between responders and non-responders to ECT in patients with TRD stratified based on intake of clonazepam.

		Unadjusted analyses		Analyses adjusted for age	
	LTL, median (IQR)	U	p	Quade's F	p
**Not treated with clonazepam**					
Responders at T1 (n=91)	0.78 (0.30)	437	0.49	0.28	0.60
Non-Responders at T1 (n=11)	0.81 (0.31)				
Responders at T2 (n=55)	0.77 (0.36)	426	0.38	0.72	0.40
Non-Responders at T2 (n=18)	0.89 (0.26)				
Remitters at T2 (n=44)	0.75 (0.30)	423	0.11	0.53	0.12
Non remitters at T2 (n=25)	0.89 (0.27)				
**Treated with clonazepam**					
Responders at T1 (n=27)	0.75 (0.26)	**10**	**0.03**	0.24	0.14
Non-Responders at T1 (n=3)	0.98 (-)				
Responders at T2 (n=10)	0.75 (0.32)	40	0.72	0.05	0.83
Non-Responders at T2 (n=9)	0.77 (0.28)				
Remitters at T2 (n= 9)	0.75 (0.30)	41	0.78	0.18	0.68
Non remitters at T2 (n=10)	0.76 (0.29)				
	Median (IQR)	Spearman's rho	p	partial corr. coeff.	p
**Not treated with clonazepam**					
Delta % MADRS T1-T0	75.34 (21.74)	-0.01	0.93	-0.06	0.54
Delta % MADRS T2-T0	80.00 (44.58)				
**Treated with clonazepam**					
Delta % MADRS T1-T0	75.53 (24.31)	**-0.36**	**0.048**	-0.23	0.23
Delta % MADRS T2-T0	73.53 (80.29)				

Abbreviations: corr. coeff, correlation coefficient; IQR, interquartile range; LTL, leukocyte telomere length; MADRS, Montgomery Asberg Depression rating scale.

Supplementary Table S4. Assessment of overrepresentation between SNPs and genes associated with response to ECT and LTL.

**Number of SNPs nominally associated with LTL**	**Number of SNPs nominally associated with response**	**Overlap between SNPs associated with LTL and response**	**p**
SNP associated with LTL: 185,410	SNPs associated with response at T1: 128,228	6,021	p = 1
	SNPs associated with response at T2: 144,770	6,222	p = 1
	SNPs associated with remission: 145,795	6,803	p = 1
	SNPs associated with Delta % T0-T1: 179,239	8,312	p = 1
	SNPs associated with Delta % T0-T2: 177,725	8,134	p = 1
**Number of genes nominally associated with LTL**	**Number of genes nominally associated with response**	**Overlap between genes associated with LTL and response**	**p**
Genes associated with LTL: 885	Genes associated with response at T1: 608	39	p = 0.11
	Genes associated with response at T2: 651	26	p = 0.93
	Genes associated with remission: 602	26	p = 0.85
	Genes associated with Delta % T0-T1: 849	45	p = 0.44
	Genes associated with Delta % T0-T2: 810	48	p = 0.17

Abbreviations: LTL, leukocyte telomere length; SNP, single nucleotide polymorphism.

Supplementary Table S5. Association between SNP previously associated with LTL and response to ECT.

				Response at T1		Delta % T0-T1		Response at T2		Remission		Delta % T0-T2		LTL in current sample	
**Chr**	**SNP**	**Closest gene**	**A1**	**OR**	**P**	**Beta**	**P**	**OR**	**P**	**OR**	**P**	**Beta**	**P**	**Beta**	**P**
1	rs3219104	*PARP1*	A	0.95	0.93	0.80	0.81	0.64	0.33	0.78	0.58	-7.37	0.33	-0.03	0.41
3	rs2613954	*RP11-572M11.4*	C	1.26	0.72	2.97	0.35	2.47	0.10	1.48	0.38	12.70	0.09	-0.02	0.60
3	rs10936600	*LRRC34 (TERC)*	T	0.55	0.21	-0.29	0.91	0.69	0.33	0.65	0.26	-2.64	0.69	0.00	0.94
4	rs60160057	*DCLK2*	A	2.33	0.26	3.80	0.18	**0.41**	**0.045**	0.42	0.06	**-16.63**	**0.02**	0.00	0.94
4	rs4691895	*NAF1*	G	0.76	0.63	0.40	0.90	2.20	0.12	1.34	0.51	-0.90	0.90	-0.02	0.68
5	rs2853677	*TERT*	G	1.20	0.71	-3.55	0.15	1.38	0.37	1.72	0.13	4.87	0.38	-0.01	0.61
6	rs2736176	*PRRC2A*	C	1.22	0.69	0.32	0.90	0.53	0.11	0.84	0.64	-9.44	0.12	-0.01	0.86
7	rs59294613	*POT1*	A	1.15	0.76	2.70	0.26	1.11	0.77	1.25	0.51	3.61	0.50	-0.02	0.47
8	rs57415150	*CSMD1*	A	1.00	1.00	-5.07	0.39	0.92	0.92	1.53	0.64	-4.57	0.75	0.00	0.97
10	rs9419958	*STN1 (OBFC1)*	T	0.47	0.13	-4.60	0.14	1.38	0.52	1.20	0.69	4.37	0.54	-0.06	0.08
11	rs228595	*ATM*	G	1.79	0.23	2.36	0.33	0.78	0.49	0.82	0.56	-2.28	0.69	0.05	0.11
12	rs7311314	*SMUG1*	A	3.45	0.06	-0.16	0.95	1.41	0.39	1.67	0.18	4.41	0.46	0.01	0.74
14	rs2302588	*DCAF4*	C	0.59	0.36	-6.87	0.05	1.28	0.64	1.09	0.86	-1.56	0.85	0.03	0.49
15	rs12909131	*ATP8B4*	T	0.61	0.38	-1.38	0.68	0.79	0.60	0.88	0.78	-7.73	0.30	0.00	0.92
16	rs3785074	*TERF2*	G	0.60	0.31	-4.29	0.13	1.26	0.60	0.95	0.90	1.35	0.84	0.00	0.92
16	rs7194734	*MPHOSPH6*	C	1.36	0.58	4.23	0.12	**0.39**	**0.032**	0.72	0.42	-13.05	0.05	**0.07**	**0.04**
18	rs2124616	*TYMS*	A	1.03	0.97	3.43	0.44	0.59	0.42	0.74	0.64	-2.49	0.82	0.01	0.88
19	rs8105767	*ZNF208*	G	0.44	0.07	**-7.19**	**0.003**	0.73	0.41	0.49	0.07	-11.26	0.07	0.01	0.78
20	rs75691080	*STMN3*	T	1.39	0.69	0.60	0.88	2.00	0.27	3.37	0.05	13.68	0.13	0.01	0.76
20	rs932827	*ZBTB46*	T	0.85	0.76	2.30	0.43	0.72	0.46	0.60	0.25	-0.79	0.91	-0.04	0.29
20	rs73624724	*ZBTB46*	C	*NA*	*NA*	0.99	0.86	0.29	0.09	0.32	0.13	-19.26	0.12	0.07	0.31

Abbreviations: A1, effect allele; Chr, chromosome; LTL, leukocyte telomere length; NA, not available, OR, odds ratio; SNP, single nucleotide polymorphism.
